# Optimized extraction technology of glutathione from ‘Haidao 86’ germ rice by response surface methodology

**DOI:** 10.1002/fsn3.3651

**Published:** 2023-09-01

**Authors:** Shengquan Ye, Junmei Huang, Wenlong Wu, Jianping Chen, Saiyi Zhong, Guang Qiu, Wei Zhang, Ru Chen, Yijun Liu

**Affiliations:** ^1^ Guangdong Provincial Key Laboratory of Aquatic Product Processing and Safety, College of Food Science and Technology Guangdong Ocean University Zhanjiang China; ^2^ Zhanjiang Yan Tang Dairy Co., Ltd. Zhanjiang China; ^3^ Hainan Key Laboratory of Storage & Processing of Fruits and Vegetables, Agricultural Products Processing Research Institute Chinese Academy of Tropical Agricultural Sciences Zhanjiang China

**Keywords:** extraction, germ powders, glutathione, Haidao 86, response surface methodology

## Abstract

Glutathione is an important functional component of ‘Haidao 86’, which has many important physiological functions in organisms and is widely used in medicine and other industries. In this study, the effects of four extraction methods (hot water extraction, formic acid extraction, ethanol extraction, and sulfuric acid extraction) on the yield of glutathione in ‘Haidao 86’ germ powders were studied by high‐performance liquid chromatography, and the yield of glutathione in hot water extraction was the highest. The effects of material–liquid ratio, temperature, pH, and time on the extraction rate of glutathione from ‘Haidao 86’ were investigated by single‐factor experiment and Box–Behnken combined experiment. The results showed that the order of influence on GSH yield was pH, temperature, material–liquid ratio, and time, and the interaction of extraction time and pH had a significant influence on glutathione yield of ‘Haidao 86’ germ powders. The optimum parameters for hot water extraction of glutathione from ‘Haidao 86’ germ powders were determined as follows: material–liquid ratio of 1:12, pH value of 2.8, temperature of 84.9°C, time of 14 min, and the extraction rate of glutathione was 139.68 mg/100 g. It provided the scientific proof for the development and industrial production of functional products of ‘Haidao 86’.

## Graphical abstract

The effects of four extraction methods (hot water extraction, formic acid extraction, ethanol extraction, and sulfuric acid extraction) on the yield of GSH in ‘Haidao 86’ germ powders were studied by high‐performance liquid chromatography (HPLC), and the yield of GSH in hot water extraction was the highest. The optimum parameters for hot water extraction of GSH from ‘Haidao 86’ germ powders were determined as follows: material–liquid ratio of 1:12, pH value of 2.8, temperature of 84.9°C, time of 14 h, and the extraction rate of glutathione was 139.68 mg/100 g.

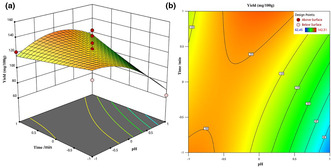

## 1 INTRODUCTION

‘Haidao 86’ is a saline‐tolerant hybrid indica rice cultivated by Chen Risheng (known as the “Father of Sea Rice”) for 33 years. Due to the presence of a large number of salt‐induced genes, sea rice can not only grow in seaside mudflats and tolerate short‐term immersion in seawater but also in deserted inland saline lands. Sea rice has become a high‐quality food germplasm resource. Compared with conventional rice, ‘Haidao 86’ not only has taller and stronger plants, but also rice that is carmine in color, rich in selenium, amino acids, flavonoids, and other nutrients, and has a sweeter taste (Du et al., 2021). In addition, ‘Haidao 86’ is a natural organic rice with fewer pests and diseases and no need to spray pesticides and chemical fertilizers (Gao et al., 2023).

Previous reports indicated that the research on sea rice mainly focused on planting, the saline‐alkali tolerance principle, yield improvement, and soil modification (Chen et al., 2017; Xie et al., 2021). The root system of ‘Haidao 86’ was promoted in low‐salt seawater (3‰), the optimum salinity for seed germination was 3‰, and the maximum salinity tolerance reached 15‰ (Sun et al., 2022). Experiments in Xinjiang, Heilongjiang, Guangdong, and other places were successful, among which the unit yield of sea rice in the Xinjiang Kashi saline‐alkali soil experimental field reached 8.24 tons/ha (Wang et al., 2019), and the unit yield of sea rice in the Qingdao saline‐alkali soil experimental field reached 10.38 tons/ha (Zhang, 2018, 2022). China had more than 100 million hectares of saline‐alkali land, of which 18 million hectares could be utilized (Wang et al., 2022), and the ‘Haidao 86–cotton’ intercropping cultivation model could be adopted to further improve the application prospect of ‘Haidao 86’ (Zhao et al., 2019). According to the calculation of 3000–4500 kg/ha, ‘Haidao 86’ will increase grain production by tens of billions of kilogram, providing more grain for mankind.

Sea rice contained 8.16% crude protein, 2.36% crude fat, 71.18% carbohydrate, and functional components such as inositol 6‐phosphate (Lai et al., 2022). Experimental studies had found that environmental stress caused sea rice cells to produce stress reactions and produce various bioactive substances such as glutathione (GSH), γ‐aminobutyric acid, and IP6 (Liang et al., 2018; Mark & Romola, 2003). GSH was a tripeptide containing γ‐amide bond and a sulfhydryl group, which was composed of glutamic acid, cysteine, and glycine. GSH could help maintain normal immune system function and had antioxidant and detoxification functions (Hristov, 2022). The sulfhydryl group on cysteine was its active group, which was easy to combine with some drugs and toxins so that it had an integrated detoxification effect. GSH could be used not only in medicine but also as the base material of functional foods, which were widely used for purposes such as delaying aging, enhancing immunity, and resisting tumors (Pei et al., 2023). At present, natural glutathione is mainly derived from soybean embryos, wheat embryos, corn embryos, etc. (Li, Xu, & Dong, 2015). However, the studies had found that the content of GSH in the embryo of ‘Haidao 86’ was very high, which provided a new way to obtain GSH. GSH (often referred to as reduced glutathione) is an acidic peptide with an isoelectric point of 5.93. It was stable under acidic conditions (Zare et al., 2023) and soluble in polar solvents such as water, dilute alcohol, and dilute acid (De et al., 2023). The common extraction methods of GSH were formic acid, ethanol, sulfuric acid, hot water extraction, and other extraction methods (Altemimi et al., 2023; Chen et al., 2009; Li, Wang, & Tian, 2015; Wang et al., 2020, 2021), and the content of seed germ could reach 100–1000 mg/100 g (Wang et al., 2021). The retrieval results showed that there was no report on the extraction of GSH from ‘Haidao 86’. In this study, the effects of four kinds of extraction methods (hot water extraction, formic acid extraction, ethanol extraction, and sulfuric acid extraction) on the GSH yield of ‘Haidao 86’ were compared by high‐performance liquid chromatography (HPLC). The effects of material–liquid ratio, temperature, pH, and time on the GSH yield of ‘Haidao 86’ were studied by single‐factor experiment and Box–Behnken combined experiment, which provided technical support for the deep processing of ‘Haidao 86’.

## 2 MATERIALS AND METHODS

### 2.1 Preparation of ‘Haidao 86’ germ rice powder

‘Haidao 86’ was purchased from Guangdong Haidao Biotechnology Co., Ltd. The preparation of germ rice flour referred to the method of Chen et al. (2018) with some modifications appropriately. Ten kilograms of ‘Haidao 86’ was soaked in water at 30°C for 10 h, filtered to dryness, and cultured in an incubator at 30°C (SPX‐150B‐Z, Qingdao Mingbo Environmental Protection Technology Co., Ltd.) for 12 h. Germ rice with a germ length of 0.5–1.0 mm was dried in a hot air‐drying oven (DHG‐9140a type, Shanghai Yiheng Scientific Instruments Co., Ltd.) at 60°C for 24 h, and then crushed by an ultra‐micro pulverizer (FDV air‐induced, Taiwan Province Hongquan Machinery) for 15 min, and passed through 100 meshes to obtain ‘Haidao 86’ germ rice powder (HDGRP).

### 2.2 Extraction of GSH

The effects of four solvent extraction methods, namely formic acid, ethanol, sulfuric acid, and hot water, on the extraction rate of GSH in HDGRP were investigated. Ten grams of HDGRP was placed in a 1‐L beaker, and 9% formic acid, 40% ethanol, 4% sulfuric acid, and distilled water at 90°C were added with a material‐to‐liquid ratio of 1:9, respectively. After stirring and extracting for 15 min, the sample was quickly cooled in an ice water bath, centrifuged at 4°C with a freezing centrifuge (Sigma 3–18 K, Sigma Laborzentrifugen GmbH) at 16,700 *g* for 5 min, and the supernatant was taken out. The supernatant was ultrafiltered by a polyethersulphone (PES) membrane with a membrane pore size of 0.1 μm, and formic acid extract, ethanol extract, sulfuric acid extract, and hot water extract were obtained, respectively. The extracts were stored at 4°C for testing.

### 2.3 Determination of GSH

Establishment of the GSH standard curve: a 5.0 mg of GSH standard was accurately weighed, dissolved in deionized water, and put in a 50‐mL volumetric flask, and the concentration of GSH solution was 100.0 μg/mL. 0.0, 5.0, 10.0, 20.0, 40.0, 80.0, and 100.0 mL of GSH solutions with a concentration of 100.0 μg/mL were transferred into a 250‐mL volumetric flask to prepare GSH standard solutions with different concentrations. The standard curve equation of different concentrations of GSH standard solution (*c*, μg/mL) and chromatographic peak area (*A*) was established, where *A* = 13.13294 × *c* + 5.00915.

Determination of GSH: The content of GSH in HDGRP was measured by HPLC, referring to the method of Xu et al. (2021) The high‐performance liquid chromatograph (Agilent1260, Agilent Company) was equipped with a DAD diode array detector and an Agilent Eclipse XDB‐C18 chromatographic column (5 μm, 4.6 × 150 mm). A sample of 5 μL was pumped onto the column; the column temperature was 30°C and the ultraviolet detection wavelength was 210 nm. Gradient elution was performed using phase A (0.1% formic acid) and phase B (methanol) with elution conditions of 98% A and 2% B for 6 min, 40% A and 60% B for 9 min, and 98% A and 2% B for 5 min at a rate of 0.5 mL/min.

The yield of GSH (*Y*) was calculated according to the following formula: *Y* (mg/100 g) = 100 × *c* × *v*/*m*, where *c*, *v*, and *m* represent the concentration of sample solution (mg/mL), the total volume of sample solution (mL), and the mass of raw materials (g), respectively.

### 2.4 Single factor experiments on hot water extraction of GSH

Effect of material–liquid ratio on the yield of GSH: the HDGRP was put into a 2‐L beaker and distilled water was added with the material–liquid ratios of 1:10, 1:12, 1:15, 1:20, 1:25, 1:30, and 1:60, respectively, and the pH extract solution was adjusted to 3 by adding hydrochloric acid with a concentration of 1 mol/L, and mixed and placed quickly in a 90°C oscillating constant‐temperature water bath for 15 min. The cooling liquid was centrifuged at 4°C for 5 min at 16,700 *g*, and the supernatant was ultrafiltered with a PES membrane with a pore size of 0.1 μm and the GSH was determined according to the method described in Section 2.3.

Effect of pH on the yield of GSH: the HDGRP was put into a 2‐L beaker, and distilled water was added with a material–liquid ratio of 1:12, and the pH extract solution was adjusted with 1 mol/L hydrochloric acid to 1, 2, 3, 4, 5, 6, and 7, respectively, and mixed and placed quickly in a 90°C oscillating constant‐temperature water bath for 15 min. The cooling liquid was centrifuged in a centrifuge at 4°C for 5 min at 16,700 *g*, and the supernatant was ultrafiltered with a PES membrane with a membrane pore size of 0.1 μm. The GSH was determined according to the method described in Section 2.3.

Effect of temperature on the yield of GSH: the HDGRP was put into a 2‐L beaker, and distilled water was added with a material–liquid ratio of 1:12, and the pH extract solution was adjusted to 3 by adding hydrochloric acid with a concentration of 1 mol/L, and mixed and placed quickly in a vibrating constant‐temperature water bath at 25, 35, 45, 55, 65, 75, 85, 95, and 105°C for 15 min. The cooling liquid was centrifuged in a centrifuge at 4°C for 5 min at 16,700 *g*, and the supernatant was ultrafiltered with a PES membrane with a membrane pore size of 0.1 μm. The GSH was determined according to the method described in Section 2.3.

Effect of time on the yield of GSH: the HDGRP was put into a 2‐L beaker, and distilled water was added with a material–liquid ratio of 1:12, and the pH extract solution was adjusted to 3 by adding hydrochloric acid with a concentration of 1 mol/L, and mixed and placed quickly in a 90°C oscillating constant‐temperature water bath for 2, 4, 6, 8, 10, 12, 14, 16, 18, and 20 min, respectively. The cooling liquid was centrifuged in a centrifuge at 4°C for 5 min at 16,700 *g*, and the supernatant was ultrafiltered with a PES membrane with a membrane pore size of 0.1 μm. The GSH was determined according to the method described in Section 2.3.

### 2.5 Single‐factor experiments on hot water extraction of GSH

According to the results of the single‐factor experiment, with the material–liquid ratio, temperature, pH, and time as independent variables and the yield of GSH as the response value, the optimum technological parameters of GSH hot water extraction were optimized by using the central composite design principle of Box–Behnken in Design‐Expert 8.0.6. The factor‐ level design is shown in Table 1.

**TABLE 1:** Levels design of test factors.

Factors	Code	Levels		
		−1	0	1
Material–liquid ratio (g/mL)	*A*	1:20	1:15	1:10
Temperature (°C)	*B*	75	85	95
pH	*C*	2	3	4
Time (min)	*D*	10	12	14

### 2.6 Data processing and analysis

Microsoft Office Excel (2010, Microsoft Corporation) and OriginPro (2021, OriginLab Corporation) was used for data analysis and plotting, and Design‐Expert 8.0.6 was used to design response surfaces, analyze variance, and predict the best process conditions.

## 3 RESULTS AND ANALYSIS

### 3.1 Effects of four extraction methods on the yield of GSH in HDGRP

**FIGURE 1:**
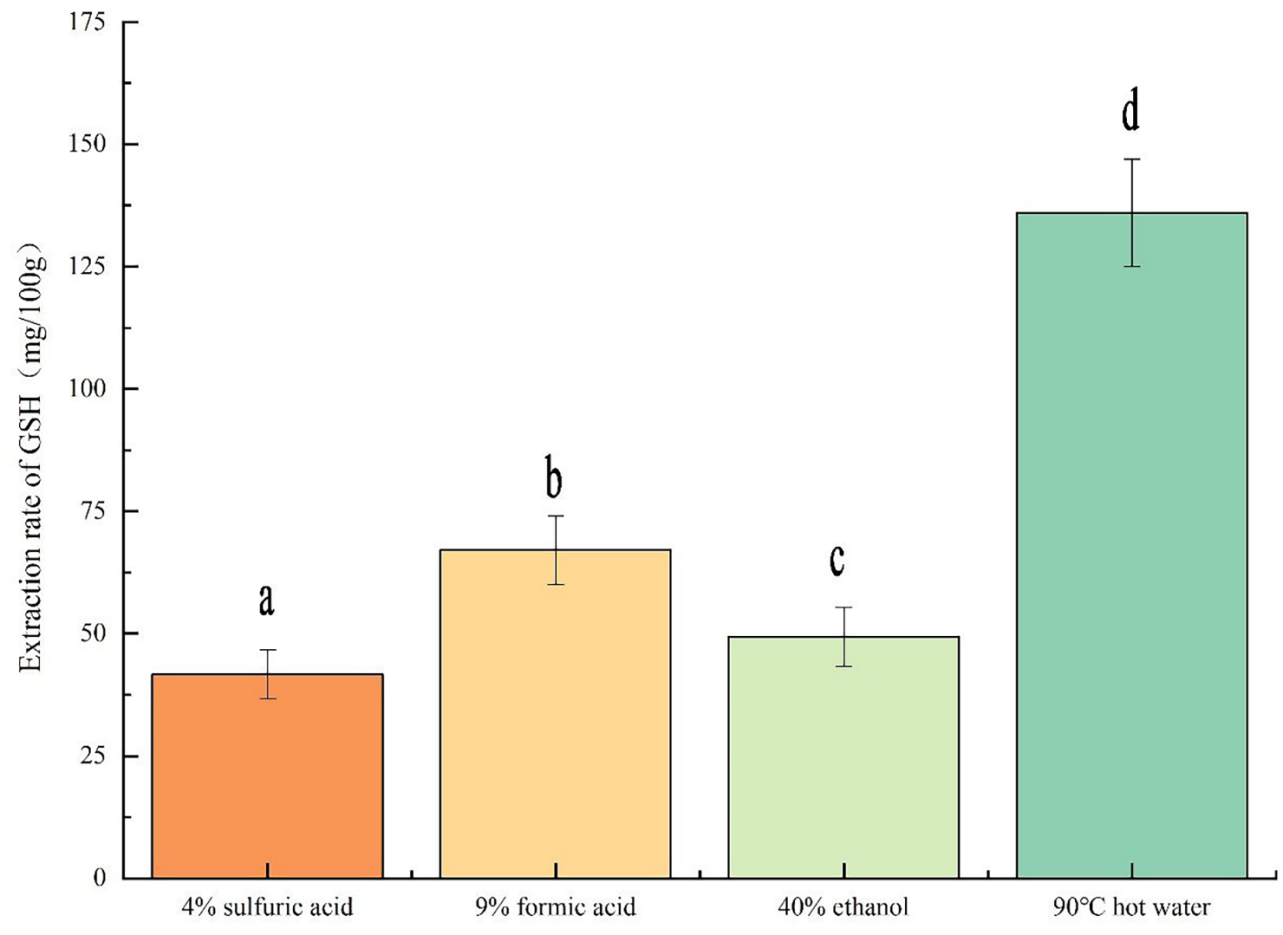
Effects of four extraction methods on the yield of GSH in HDGRP. Different letters (a–d) represent significant differences.

The effects of four extraction methods (90°C hot water extraction, 9% formic acid extraction, 40% ethanol extraction, and 4% sulfuric acid extraction) on the yield of GSH in HDGRP are shown in Figure 1. It could be seen from Figure 1 that the four extraction methods had significant effects on the extraction rate of GSH in HDGRP, and the yield of GSH obtained by hot water extraction was the highest (135.98 ± 11.20 mg/100 g), which was consistent with the research results of Zhang (2015) The reason for this result was related to the property that GSH was easily soluble in water and insoluble in ethers and alcohols. Therefore, it was important to optimize the technological parameters of the hot water extraction of HDGRP in the next experiment.

### 3.2 Effects of different extraction conditions on the yield of GSH in HDGRP

**FIGURE 2:**
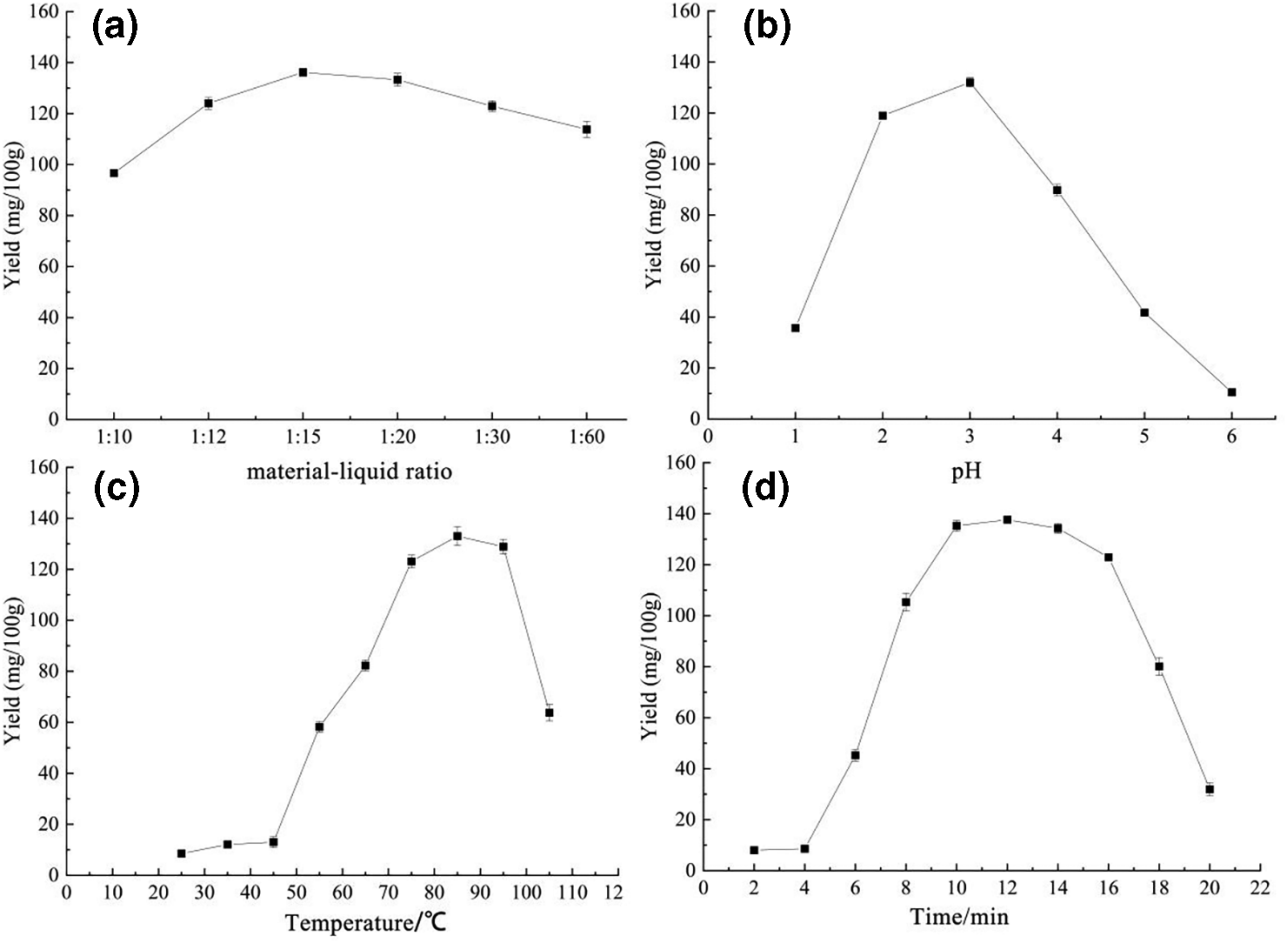
(a–d) Respectively, represent the effects of material–liquid ratio, pH, temperature, and time on the yield of GSH in HDGRP.

The effects of different material–liquid ratios, pH, temperature, and time on the yield of GSH in HDGRP during hot water extraction are shown in Figure 2. From Figure 2a, with the increase of the material–liquid ratio, the yield of GSH first increased and then decreased, and the maximum yield of GSH was 136.17 ± 1.56 mg/100 g at the material–liquid ratio of 1:15. When the ratio of material‐to‐liquid was greater than 1:20, the exposed area of low concentration samples increased and it was easy to be oxidized, which led to a decrease in GSH yield (Pei et al., 2023). When the ratio of material to liquid was less than 1:12, the fluidity of the extract was low and the dissolution efficiency of GSH was low, which led to a decrease in GSH yield. When the pH was between 2 and 4, the yield of GSH was above 200 mg/100 g. GSH was an acidic tripeptide with an isoelectric point of 5.93 (Zhang, 2015). Near the isoelectric point, the solubility and dissolution rate of GSH were low, so the yield was reduced, and when pH was lower than 2, the GSH extraction rate decreased rapidly, so it was difficult to realize large‐scale production. From Figure 2c, with the increase of temperature, the permeability of cell wall increases, the mass transfer rate accelerates, and the yield of GSH first increases and then decreases sharply. When the temperature was above 95°C, the dissolution of GSH was accelerated, and the chances of contact with oxygen in air and water were increased, which led to more reduced glutathione being transformed into oxidized glutathione (GSSG), and the yield was down. From Figure 2d, with the increase in time, the yield of GSH increased first and then decreased, and the maximum yield of GSH was 137.60 ± 1.05 mg/100 g at 12 min. Glutathione, an intracellular peptide, was slowly and gradually extracted from cells. When the extraction time was less than 12 min, the yield of glutathione increased with the increase in time. The longer the extraction time, the more oxidation there is and the lower the yield.

### 3.3 Response surface method test results

#### 3.3.1 Box–Behnken design experiment and regression model

According to the design principle of the Box–Behnken experiment, four factors, namely the material–liquid ratio (*A*), pH (*B*), temperature (*C*), and time (*D*), were selected to carry out 29 experiments on the extraction technology of GSH from HDGRP. The experimental results and variance analysis are shown in Tables 2 and 3, respectively. From Table 3, the *p*‐value of .0001 indicated that the model was of great significance. The *p*‐value of .2239 implied that the lack of fit was not significant relative to pure error. The correlation coefficient *R* ^2^ of .9107 showed that the model could explain the change of 91.07% response value, and the quadratic model could be used to predict the yield of GSH extracted by hot water extraction under different factors.

**TABLE 2:** Box–Behnken design and results.

No.	*A* (g/mL)	*B* (°C)	*C* (pH)	*D* (min)	*Y* (mg/100 g)
1	0	0	0	0	133.09
2	0	0	0	0	125.66
3	−1	1	0	0	95.97
4	−1	0	0	−1	105.25
5	1	1	0	0	120.53
6	1	0	0	1	128.20
7	−1	−1	0	0	74.48
8	−1	0	0	1	103.24
9	0	1	−1	0	120.55
10	0	−1	−1	0	74.80
11	0	0	0	0	142.31
12	0	1	0	1	100.22
13	0	0	1	−1	63.71
14	0	−1	0	1	96.14
15	0	1	0	−1	95.02
16	0	0	−1	1	121.46
17	0	0	0	0	122.08
18	1	0	0	−1	97.11
19	0	−1	0	−1	90.29
20	0	0	0	0	125.40
21	0	−1	1	0	62.45
22	1	0	−1	0	124.90
23	1	0	1	0	109.71
24	0	0	1	1	123.18
25	1	−1	0	0	84.45
26	−1	0	1	0	71.41
27	0	0	−1	−1	119.97
28	−1	0	−1	0	108.64
29	0	1	1	0	71.45

**TABLE 3:** ANOVA for quadratic model.

Source	Sum of squares	df	Mean square	*F*‐value	*p*‐Value	Significant
Model	33.03	14	2.36	9.96	<.0001	Significant
*A*‐*A*	2.32	1	2.32	9.80	.0074	*
*B*‐*B*	3.29	1	3.29	13.92	.0022	*
*C*‐*C*	6.51	1	6.51	27.51	.0001	*
*D*‐*D*	2.18	1	2.18	9.23	.0089	*
AB	0.0969	1	0.0969	.4094	.5326	
AC	0.4039	1	0.4039	1.71	.2126	
AD	0.6135	1	0.6135	2.59	.1298	
BC	0.7926	1	0.7926	3.35	.0887	
BD	0.0004	1	0.0004	.0017	.9679	
CD	2.32	1	2.32	9.82	.0073	*
*A* ^2^	1.43	1	1.43	6.06	.0274	**
*B* ^2^	11.90	1	11.90	50.24	<.0001	*
*C* ^2^	4.85	1	4.85	20.48	.0005	*
*D* ^2^	0.7725	1	0.7725	3.26	.0924	
Residual	3.31	14	0.2368			
Lack of fit	2.82	10	0.2817	2.26	.2239	Not significant
Pure error	0.4978	4	0.1244			
Cor total	36.34	28				

* Represents the extreme significance of the model (*p* < .01),

** Represents the significance of the model (*p* < .05).

According to the 2*F*‐value in Table 3, the degree of influence of various factors on the yield of GSH was pH > temperature > material–liquid ratio > time. The interaction between pH and time, the first term of material–liquid ratio, pH, temperature, and time, and the second term of material–liquid ratio, temperature, and pH also had a significant influence on the yield of GSH in HDGRP. The regression equation of glutathione yield (*Y*) in HDGRP was obtained by eliminating the insignificant items in the model, that is, *Y* = 11.1607 + 0.4396 × *A* + 0.5240 × *B* − 0.7367 × *C* + 0.4267 × *D* + 0.7622 × *C* × *D* − 0.4050 × *A* ^2^ − 1.2889 × *B* ^2^ − 0.7993 × *C* ^2^.

#### 3.3.2 Response surface analysis of GSH yield of HDGRP

**FIGURE 3:**
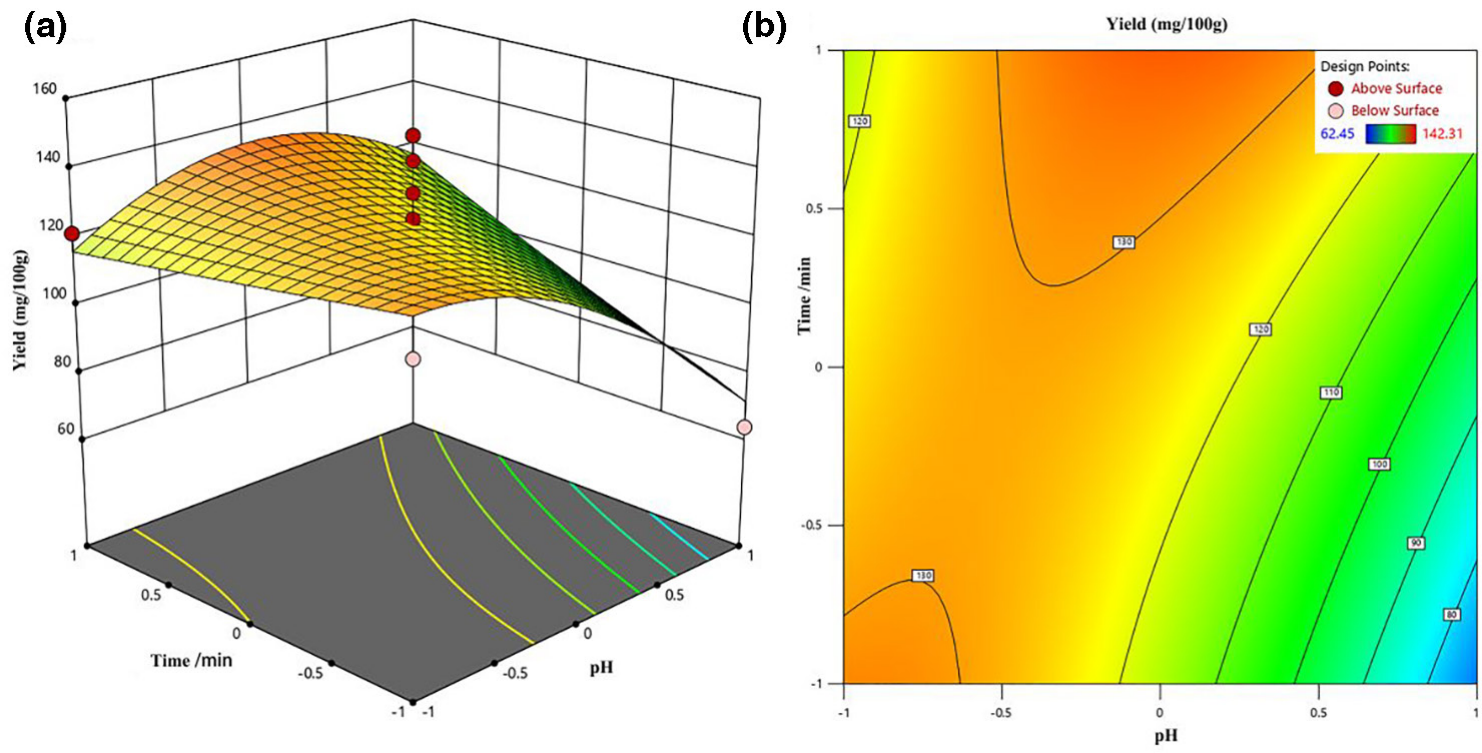
(a, b) Represent the response surface and contour map of time and pH to GSH yield, respectively.

According to the regression equation, the three‐dimensional curved surface diagram of GSH yield and experimental factors was established, and the optimal conditions for the maximum GSH yield were determined. It could be seen from Table 3 that the interaction between pH and time was significant (*p* < .05), while the interaction of other factors was not obvious (*p* > .05). The response surface and contour map of the interaction between pH and time are shown in Figure 3 with a material‐to‐liquid ratio of 1:15 and a temperature of 85°C. From Figure 3a,b, the interaction surface between time and pH was inverted *U*‐shaped, and the GSH extraction rate was the highest at the apex. When the pH value was between 3 and 4, the contour lines on the curved surface were dense and steep, which indicated that the pH value had a great influence on the extraction rate of GSH, and the higher the pH value, the lower the extraction rate of GSH. The pH was between 2 and 3, and the contour lines on the curved surface were sparse and smooth, which showed that the pH has little influence on the extraction rate of GSH in this range, which showed that too high and too low pH values were not conducive to the extraction of GSH. When the extraction time was in a different pH range, the degree of curvature of the surface was different, which had different effects on the extraction rate.

#### 3.3.3 Calculation of optimal extraction parameters

According to the regression model of material–liquid ratio, pH, temperature, time, and GSH yield, the optimum extraction parameters of hot water extraction of GSH from HDGRP were determined and calculated as follows: the material–liquid ratio of 1:12.30, pH value of 2.797, temperature of 84.85°C, time of 14 h, and the predicted extraction rate of glutathione was 138.601 mg/100 g. In order to facilitate the control of experimental parameters, the parameters obtained by calculation and optimization were readjusted, and the material–liquid ratio, pH, temperature, and time were 1:12, 2.8, 84.9°C, and 14 h, respectively. Under the optimum extraction conditions, the verification experiment was carried out, and the optimal result determined by three experiments was 139.68 mg/100 g, which showed the rationality of the optimization result and further verified the applicability of the regression model.

## 4 DISCUSSION

Natural glutathione was mainly extracted from plant seeds, yeast, and chicken blood, among which cereal germ was a good source of glutathione. The content of GSH from different sources was quite different, with 51.55 mg/100 g of glutathione from wheat germ (Li, Xu, & Dong, 2015), 145 mg/100 g from corn germ (Zare et al., 2023), 8.8213 mg/100 g from wine mud (De et al., 2023), and 29.94 mg/100 g from yeast (Roswag et al., 2022). In this study, the GSH content of HDGRP (up to 139.68 mg/100 g) was higher than that of wheat germ and yeast and slightly lower than that of corn germ. Therefore, HDGRP had important development value and could provide sufficient raw materials for the source of GSH.

Solvent extraction, chemical synthesis, enzyme synthesis, biological fermentation, and other methods were commonly used to extract GSH (GeorgiouSiafis et al., 2022; Jin et al., 2022; Wang et al., 2021), although chemical synthesis had been eliminated by industrialization because of its low purity and unstable titer. The enzymatic synthesis method had high specificity and high product purity, but its cost was high. The extraction method for producing GSH had the advantages of mature method, simple operation, low technological requirements, less pollution, and high product purity, which made it convenient for industrial production. In this study, the effects of four solvent extraction methods, formic acid, ethanol, sulfuric acid, and hot water, on the GSH yield from HDGRP were compared. The hot water extraction method had the highest GSH yield, which was consistent with the research results of Zhang (2015). Hot water extraction method takes advantage of the characteristics of glutathione's low molecular weight and good water solubility to efficiently extract glutathione from cells under the condition of incomplete wall breaking. Adjusting the pH value could improve the extraction efficiency, reduce the oxidation loss of the sample, effectively reduce the content of macromolecular peptides in the sample, and reduce the loss of equipment such as membrane filtration. Hot water extraction will be a good choice for industrial production in the future.

GSH has important physiological functions in organisms. It maintains the redox environment in the body by controlling the redox potential; protects the sulfhydryl surface in the body from being damaged by oxidants by transferring sulfhydryl groups, combining with harmful substances in the body, eliminating the combination, and detoxifying (GeorgiouSiafis et al., 2022); and assists in treating diseases related to human diseases such as Parkinson's disease, AIDS, liver disease (Zhu et al., 2023), and GSH metabolism changes by participating in cofactors of various cell enzymes in the body (Tanzilli et al., 2021). However, GSH has reduced glutathione (GSH) and oxidized glutathione (GSSG), and GSH plays a major physiological function in organisms. It had been reported that GSH was easily oxidized into GSSG in water, but when the isoelectric point of GSH was 2–3, reduced GSH was dominant and had the best stability (Gotoh et al., 2003). Therefore, properly controlling the pH value of the extract could not only improve the yield of GSH but also improve its activity and stability.

## 5 CONCLUSIONS

Compared with hot water extraction, formic acid extraction, ethanol extraction, and sulfuric acid extraction, the yield of glutathione in hot water extraction was the highest. According to the results of the single‐factor experiments, a quadratic regression model was established by using the Box–Behnken central composite design principle and response surface methodology to determine the best hot water extraction conditions for GSH from HDGRP. The optimum parameters for hot water extraction were determined as follows: material–liquid ratio of 1:12, pH value of 2.8, temperature of 84.9°C, time of 14 min, and the extraction rate of glutathione was 139.68 mg/100 g. In this study, reduced glutathione was extracted from the broken germ powder of ‘Haidao 86’. Natural glutathione was expensive and in short supply. If ‘Haidao 86’ could be planted on a large scale, GSH products could be industrialized.

## AUTHOR CONTRIBUTIONS

**Shengquan Ye:** Conceptualization (equal); funding acquisition (equal); resources (equal); writing – original draft (equal). **Junmei Huang:** Conceptualization (equal); supervision (equal); writing – review and editing (equal). **Wenlong Wu:** Methodology (equal); project administration (equal); validation (equal). **Jianping Chen:** Formal analysis (equal); investigation (equal); project administration (equal). **Saiyi Zhong:** Methodology (equal); project administration (equal); resources (equal); writing – original draft (equal). **Guang Qiu:** Formal analysis (equal); funding acquisition (equal); investigation (equal); resources (equal). **Wei Zhang:** Conceptualization (equal); funding acquisition (equal); supervision (equal); validation (equal). **Ru Chen:** Conceptualization (equal); data curation (equal); resources (equal). **Liu Yijun:** Conceptualization (equal); formal analysis (equal); software (equal); writing – original draft (equal); writing – review and editing (equal).

## FUNDING INFORMATION

This work was funded by the Research and Development of Industrialization of Sea Rice‐Dairy Products (B20083), the Guangdong Ocean University Innovation Program (230419100), the Nanhai Youth Scholar Project of Guangdong Ocean University (002029002009), the Project of Science and Technology of Zhanjiang City (2020A01034), and the Innovative Team Program of High Education of Guangdong Province (2021KCXTD021).

## CONFLICT OF INTEREST STATEMENT

The authors declare that they have no known competing financial interests or personal relationships that could have appeared to influence the work reported in this paper.

## Data Availability

Data are contained within the article.
